# The Effect of *Commiphora molmol* Nanoparticles as an Endodontic Irrigant on the Morphology, Viability, Migration, and Proliferation of Human Bone Marrow Mesenchymal Stem Cells: An In Vitro Study

**DOI:** 10.3390/ijms26041412

**Published:** 2025-02-07

**Authors:** Sultanah AlMobarak, Ebtissam AlMadi, Amal Almohaimede, Mohamed Badran, Rhodanne A. Lambarte

**Affiliations:** 1 Department of Restorative Dental Sciences, Endodontic Division, College of Dentistry, King Saud University, Riyadh 11432, Saudi Arabia; aalmohaimede@ksu.edu.sa; 2 King Abdullah Bin Abdulaziz University Hospital, Princess Nourah University, Riyadh 13412, Saudi Arabia; 3 Department of Pharmaceutics, College of Pharmacy, King Saud University, Riyadh 11495, Saudi Arabia; mbadran@ksu.edu.sa; 4 Nanobiotechnology Unit, College of Pharmacy, King Saud University, Riyadh 11495, Saudi Arabia; 5 Molecular and Cell Biology Laboratory, Prince Naif bin AbdulAziz Health Research Center, King Saud University Medical City, Riyadh 12372, Saudi Arabia; rlambarte@ksu.edu.sa

**Keywords:** *Commiphora molmol*, cytotoxicity, endodontic treatment, herbal, irrigation, myrrh, stem cells, viability

## Abstract

This study aimed to evaluate the effect of a newly formulated *Commiphora molmol* (CM) nano-irrigant on the morphology, viability, proliferation, migration, and wound healing of human bone marrow-derived mesenchymal stem cells (hBMMSCs). Different concentrations of CM nano-irrigant were prepared. The minimum inhibitory concentration (MIC) and minimal bactericidal concentration (MBC) were determined to be 25 and 30 mg/mL, respectively. The solution was dispersed into liposomes, which were subsequently coated with chitosan-forming chitosomes. Three concentrations of CM chitosomes were evaluated (25, 30, and 35 mg/mL) along with positive (5.25% NaOCl) and negative (basal culture media) control groups. Cellular viability and proliferation were quantified using AlamarBlue, while wound-healing ability was determined using the scratch assay, and 3D cellular migration was evaluated using the transwell migration assay. All tested concentrations induced observable changes in cellular morphology without any detrimental effects. Viability was monitored at 1, 6, and 24 h, with only Group 1 (25 mg/mL) showing no significant effect on cellular viability. Cellular proliferation was observed over 14 days, with Group 3 (35 mg/mL) being the only group that showed a significantly slower proliferative rate. All tested concentrations resulted in significant differences in transwell migration compared to the negative control. Significant differences were observed within each group across different time points (24–48 h). The results confirm the biocompatibility of the newly formulated CM nano-irrigant in terms of hBMMSCs’ viability, proliferation, morphology, migration, and wound healing.

## 1. Introduction

The success of endodontic treatment strongly relies on the eradication of micro-organisms that cause apical periodontitis (AP) within the root canal system (RCS). The microbial consortium within the RCS may include bacteria, viruses, fungi, and archaea [1]. However, the most common micro-organisms causing AP are bacteria [2]. Certain bacterial species possess virulent factors that aid their persistence and survival, making it harder to eliminate disease and succeed in endodontic treatment [3,4]. Sodium hypochlorite (NaOCl) has been the main root canal disinfecting agent [5]. An important advantage of NaOCl over other disinfectants is its ability to dissolve necrotic tissues [5,6], as it can host anaerobes and lead to treatment failure [7]. NaOCl has also proven effective against resistant bacterial strains [8,9,10]. The high pH of NaOCl is one of the factors contributing to its potent antimicrobial activity and tissue-dissolving ability. Most of the compounds described as successful in reducing bacterial loads in the literature have a high pH [11].

NaOCl’s stronger eradication of microbes is beneficial for achieving the primary goal of disease elimination. However, certain drawbacks have been documented [12,13,14,15]. Even though necrotic tissue dissolution is a main favorable property of sodium hypochlorite, this digestive action is nonspecific and impacts vital tissues [12,13,14,16,17]. The main concern is that its contact with vital tissues can trigger inflammation and subsequent necrosis [12,13,14,16,17]. As the RCS is a confined space, irrigants, and disinfecting agents could move beyond the apical foramen and into the periradicular area, thus compromising the viability of apical tissues, which harbor stem cells that could be helpful in future endodontic regenerative treatments [18].

Human bone marrow mesenchymal stem cells are fibroblasts that have shown an osteogenic differentiation ability in vitro [19]. When hBMMSCs are grown in vitro under specific conditions, they can differentiate into multiple cell lineages, such as adipogenic, osteogenic, chondrogenic, and other lineages, including neurogenic lineages [19]. As with other somatic stem cells, such as hematopoietic stem cells, they can form clonogenic colonies, which explains their potential for self-renewal [19].

*Commiphora molmol* (*CM*) is a natural product widely used in medicinal research [20,21,22,23]. Its exudate, commonly known as myrrh (meaning bitter), is a resinous oleo-gum product collected from the trunk of the CM plant from the *Burseraceae* family [20,24]. It has shown good anti-inflammatory, antiulcer, antihyperlipidemic, and analgesic activities [21,25]. In dental research, CM has shown promising antimicrobial, antiplaque, anti-inflammatory, and wound-healing-promoting properties [23,24,26,27].

In the literature, studies directly comparing NaOCl to CM are limited. The antimicrobial efficacy of both substances has been evaluated [24] against *Enterococcus faecalis* (*E. faecalis*) and *Fusobacterium nucleatum* (*F. nucleatum*), and myrrh solution was shown to have considerable antimicrobial effects [24]. However, cytotoxic effects have been investigated and reported separately for both [12,15,28,29,30,31,32,33]. CM was shown not only to have some cytotoxic effects directly related to increased concentration and exposure duration [30] but also to possess relatively low cytotoxic effects against human mesenchymal stem cells [34]. When applied directly to wounds, myrrh was proven to be safe and enhanced wound healing when used at low concentrations for short durations [27]. However, longer exposure times and high concentrations exhibit harmful effects and delayed healing [27]. When myrrh was added to a synthetic dermal wound dressing, proliferated cells were shown to have similar morphology to the control group with steady proliferative rates [35]. Systemic administration of myrrh on gastric ulcer and wound injury rat models showed that the groups treated with myrrh had a significant increase in the white blood cell levels pre-injury, which was maintained post-injury and throughout the healing period [36]. The cytotoxic effects of NaOCl on vital tissues included hemolysis and ulceration, leading to tissue injury [37]. Neutrophil migration was delayed, and fibroblasts were destructed [13]. In the clinical setting, trismus, hemorrhage, extensive swelling, and facial nerve paralysis are among the most reported side effects when NaOCl is introduced to the periapical area during endodontic treatment [13].

Nanotechnology was introduced in its modern form by Richard Feynman in 1965. It was preceded by the concept of the nanometer, which was used by Richard Zsigmondy specifically to describe particle size [38]. It was not until the mid-1980s that Norio Taniguchi used the term nanotechnology to describe processes that occurred on the molecular and atomic levels with particles ranging between 1 and 100 nm in size [39]. Nanotechnology has been widely used in various fields of dentistry in various forms and for multiple aims. In restorative dentistry, for instance, antibacterial nanoparticles have been incorporated into multiple restorative resin composite materials to reduce the presence and development of micro-organism biofilms [40]. Certain restorative glass ionomer cements were enhanced using nanotechnology to make them able to release fluoride ions and, therefore prevent and reduce secondary caries [39,41]. In endodontics, silver nanoparticles (AgNPs) enhanced the antimicrobial activity of calcium hydroxide (Ca(OH)_2_) against *F. nucleatum* biofilms [42].

Chitosan (CS)-coated nanocarriers are the most commonly used effective nanocarriers [43,44]. CS is a polysaccharide derived from the exoskeleton of crab shells and other crustaceans [43,44]. It possesses antimicrobial effects due to its particle size and positive charge, yet it is known for its biological compatibility with mammalian cells [45]. By enhancing the dissemination of antibacterial compounds and successfully removing adherent and non-adherent bacterial cells, CS nanoparticles have significantly aided root canal disinfection [44,46]. When used as a coating agent, CS has been shown to improve the physiochemical properties of formulations, such as particle size, surface charge, and stability [47].

Additionally, liposomes are spherical-shaped nanocarriers [48] that have been widely used in drug delivery systems due to their biocompatibility and diverse particle size ranging from 0.02 µm up to 3 μm [49]. Surface-coated liposomes could enhance the stability of therapeutic encapsulated substances and ensure more efficient delivery to targeted sites [50]. Therefore, chitosan-coated liposomes (chitosomes) have been recognized as promising drug delivery carriers [51,52,53,54]. However, they have not yet been studied in the literature for their potential as an irrigation vehicle affecting dental stem cell reservoirs, such as dental pulp stem cells (DPSC) and stem cells of apical papilla (SCAP).

Although nanoparticles demonstrate considerable antibacterial activity, several challenges, such as toxicity and undesirable effects, need to be addressed. Therefore, this study aimed to evaluate the impact of a newly formulated nano-irrigation solution containing *Commiphora molmol* chitosomes on hBMMSCs in terms of morphology, viability, wound healing, migration, and proliferation.

## 2. Results

### 2.1. Cell Morphology and Immunofluorescence Staining

**Figure 1:**
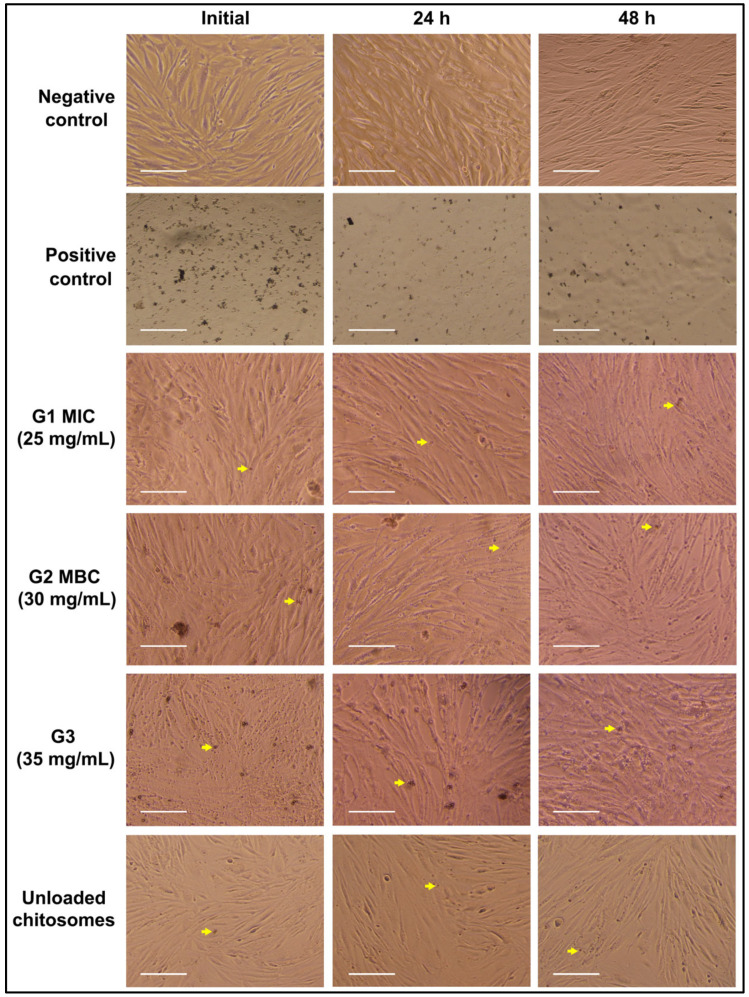
Microscopic images showing all experimental and control groups before treatment and 24 and 48 h post-treatment. All experimental groups demonstrated viable cells with minor morphological alterations. The nuclear wall and contents were observed to be intact. Unloaded chitosomes were evaluated to confirm their inertness and eliminate potential interference with the results. Yellow arrows indicate CM chitosomes.

Normal hBMMSCs showed an elongated, spindle-shaped morphology. All the experimental groups showed some changes in spindle-shaped cell morphology, with the highest concentration (35 mg/mL) causing the most morphological alteration (Figure 1). However, no damaging effects were observed. The cellular wall and nuclei were shown to be intact.

The effect of the experimental CM nano-irrigant appears to be directly correlated with its concentration. The higher-concentration group, G3 (35 mg/mL), showed the most cellular alteration even at baseline, whereas the lowest-concentration group, G1 (25 mg/mL), induced slight morphological changes (Figure 1). However, increased exposure time of up to 48 h demonstrated cellular changes among all groups.

**Figure 2:**
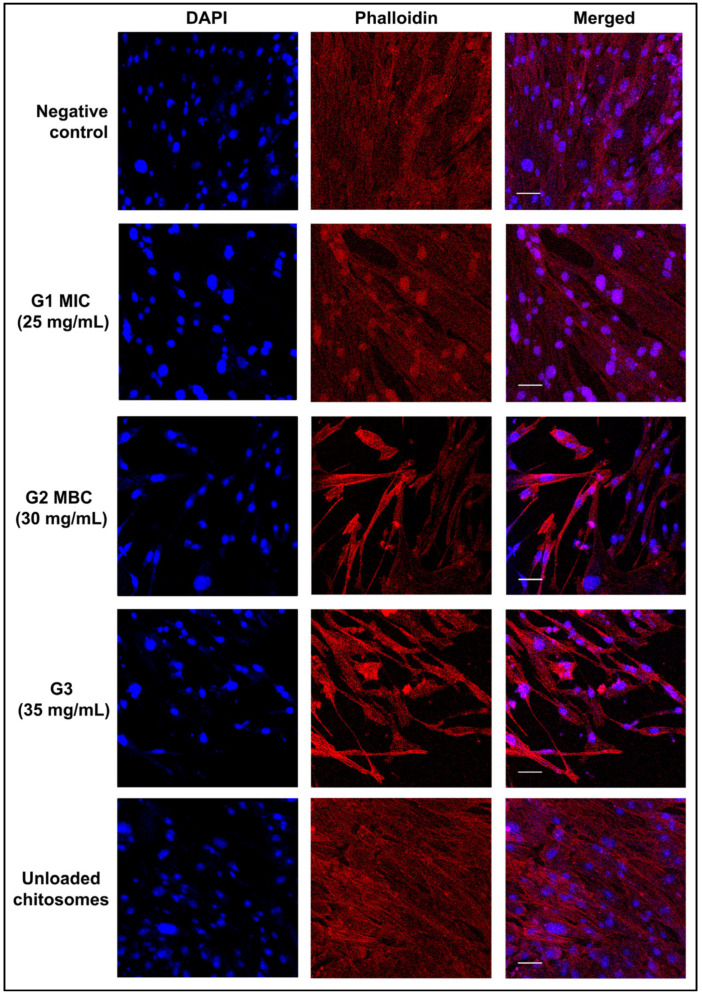
Representative images of hBMMSC groups treated with different experimental concentrations for 1 h. 4′,6-Diamidino-2-phenylindole dihydrochloride (DAPI, Invitrogen™ Molecular Probes, Eugene, OR, USA) (blue) and Alexa Fluor™ 594 Phalloidin (1:800, Invitrogen™ Molecular Probes) (red) were used to stain the nuclei and actin filaments, respectively. Images show some morphological changes while maintaining an intact nuclear wall and contents. Images from all tested concentration groups show increased densities of actin filaments and a slight reduction in cell count, with nuclei predominantly located in the central regions of the cells.

A dense extracellular matrix (ECM) and the expression of cell-mediated adhesion proteins are characteristic features of hBMMSCs. The immunofluorescence images in Figure 2 show that the least variation in morphology among the experimental groups was observed in G1 (25 mg/mL). Although all the experimental groups showed some morphological changes, none of them showed any damage to the nuclear wall or contents. All nuclei were intact and densely stained with DAPI. The actin filaments are shown in red.

### 2.2. Cell Viability and Proliferation Assay Using AlamarBlue (AB)

**Figure 3:**
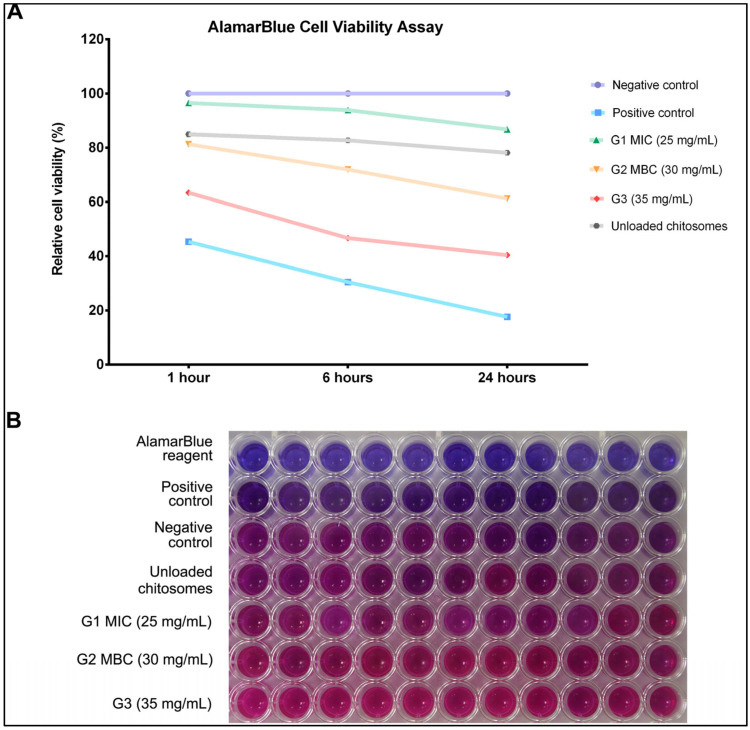
(**A**) Cell viability compared to negative control (cells without treatment) at 1, 6, and 24 h post-treatment. G1 showed the highest viability among the experimental groups at all the time points, and no significant changes were observed in the negative control group. G2 demonstrated no detrimental effects until a prolonged treatment of 24 h, wherein some significant effects on cellular viability were observed (*p* < 0.05). All values represent the means of 2 independent experiments with 11 replicates (n = 22). (**B**) Representative image of the AlamarBlue assay plate with telomerized human bone marrow-derived mesenchymal stem cell line (hMSC-TERT). Each row of wells represents the same group (labeled). Changes in medium color associated with AB reduction from blue (oxidized) to pink (reduced) with increasing irrigant concentration.

Figure 3 illustrates the cellular viability of hBMMSCs after treatment to each experimental concentration at 1, 6, and 24 h. A group of unloaded nanocarriers was also included to confirm their inert properties. The unloaded nanocarriers (chitosomes) did not result in any significant difference in cellular viability compared to the negative control group at any of the time points, demonstrating that they did not affect hMSC-TERT viability (*p* > 0.05). At 1 and 6 h, there were no significant differences in cellular viability only for G1 MIC (25 mg/mL) compared to the negative control group (untreated cells with culture media). However, significant reductions in cellular viability were observed in all groups at 24 h (*p* < 0.05). Groups 2 and 3 (30 and 35 mg/mL, respectively) showed significant reduction at all time points in comparison to the negative control group, indicating that the treatments in these groups had the greatest effects on cellular viability (*p* < 0.01). After 24 h exposure, the relative cellular viability decreased significantly to 61.22% and 40.39% at concentrations of 30 and 35 mg/mL, respectively. Upon exposure to NaOCl treatment, the hMSC-TERT cells exhibited a statistically significant decrease in cell viability immediately following treatment, with further reductions observed at both 6 and 24 h post-exposure (*p* < 0.001).

**Figure 4:**
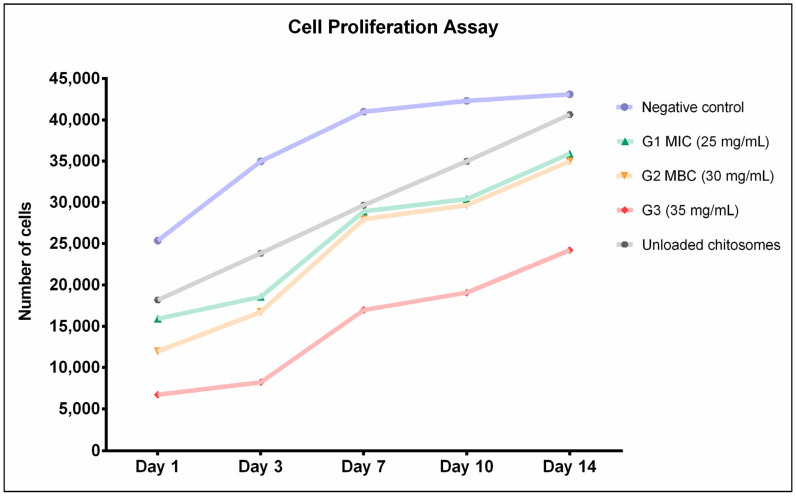
Cellular proliferation assays showed steady proliferation rates in all experimental groups, with Group 3 having the lowest cell count. Unloaded chitosomes resulted in a comparable proliferation rate to the negative control group (cells with no treatment). Data were generated from 2 independent experiments with 11 replicates (n = 22).

Cellular proliferation was monitored over a 14-day period. Observations were recorded at 1, 3, 7, 10, and 14 days. Figure 4 shows that Group 3 (35 mg/mL) had a significantly slower proliferation rate, whereas Groups 1 and 2 (25 and 30 mg/mL) displayed more rapid and consistent proliferation rates. The unloaded nanocarriers appeared to have slowed cellular proliferation but did not exert a significant effect on the final proliferative result (*p* < 0.05). All experimental groups demonstrated a trend towards increased cellular proliferation on day 7. However, significant increases in cellular proliferation were observed in all experimental groups regardless of CM nano-irrigant concentration (*p* < 0.01).

### 2.3. Transwell Migration

**Figure 5:**
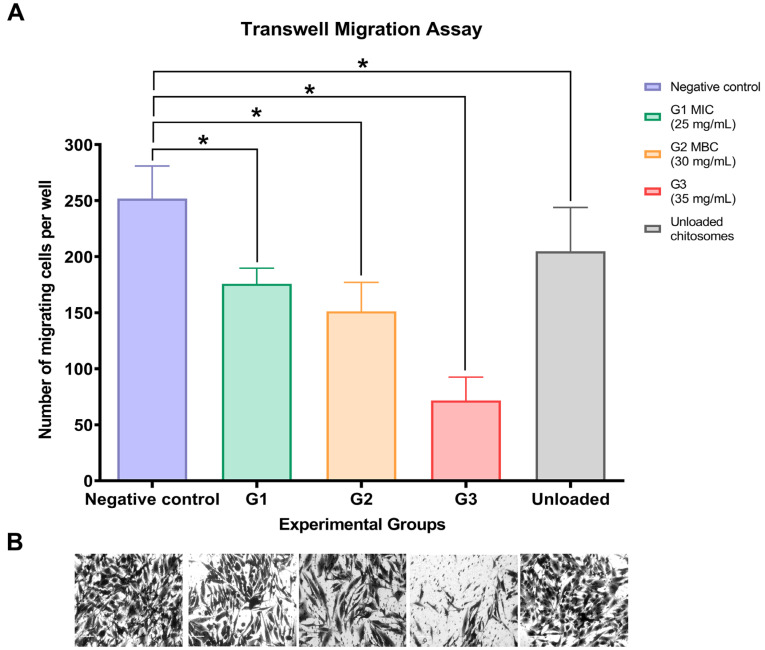
(**A**) Transwell migration assay values for experimental and control groups. All groups, including unloaded chitosomes, showed statistically significant differences compared to the negative control. (* statistical significance at *p* < 0.05). (**B**) Values represent the mean of migrating cells per well ± SD (standard deviation) of 3 independent experiments performed in triplicates (n = 9).

All experimental concentrations demonstrated an effect on the number of migrating cells in the transwell migration assay. Figure 5A illustrates significantly reduced values in all experimental groups compared to the negative control. Group 3 (35 mg/mL) resulted in the least number of migrated cells, with a mean of 71.67 cells per well. Additionally, unloaded chitosomes resulted in a mean of 204.9 migrated cells in the lower chamber of the transwell. Representative microscopic images for each group are shown in Figure 5B.

### 2.4. Wound-Healing (Scratch) Assay

**Figure 6:**
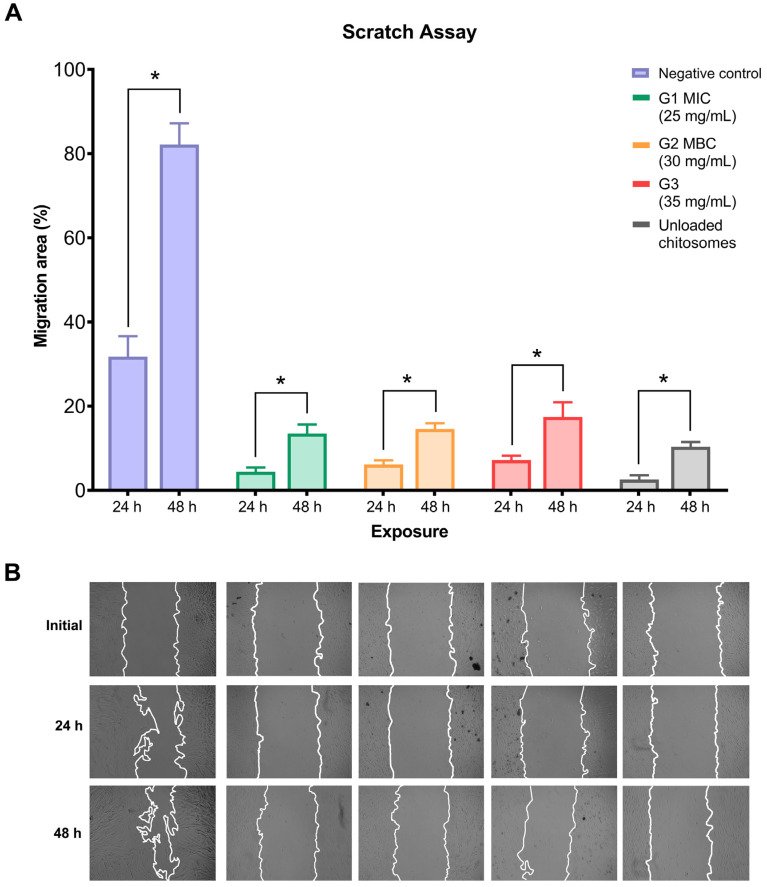
Results of the scratch migration assay at 0, 24, and 48 h. The wound closure percentage is shown in (**A**), and representative images are shown in (**B**). All groups showed significant differences post-intervention compared to initial exposure (* statistical significance at *p* < 0.05). All data were presented as mean migration area percentage ± SD of at least 4 independent experiments with triplicates (*n* = 12).

Wound closure was monitored closely for 24 and 48 h, and the results were quantified. Generally, all experimental groups showed significant positive differences within the same group in wound closure progression after 48 h compared to 24 h (Figure 6A). The negative control group appeared to have the highest percentage of wound closure among all groups at both 24 and 48 h. At the same time, unloaded chitosomes significantly decreased the migration capacity of hMSC-TERT cells compared to the untreated group. Regardless of CM nano-irrigant concentration, wound closure capabilities of hMSC-TERT cells were reduced compared with the control group. Representative images for each experimental group are presented in Figure 6B.

## 3. Discussion

Microbial eradication has always been the main goal of endodontic treatment. As micro-organisms are the main cause of endodontic pathosis, numerous disinfectants have been used in attempts to eradicate them [55,56]. Sodium hypochlorite has been the gold standard for decades [57,58,59]. In addition to its capacity to dissolve necrotic tissues, its ability to eliminate some of the most resistant organisms within the root canals has made it the most widely used disinfectant for endodontic treatment [55,60]. The most concerning drawback of NaOCl, however, is its cytotoxic effects on organic tissues. These effects can result in tissue damage and even tissue necrosis [4,5,61]. The primary concern relating to this aspect in endodontic therapy is the potential damage to dental stem cells. Such cells, specifically dental pulp stem cells (DPSCs) and stem cells of the apical papilla (SCAPs), could be beneficial in regenerative therapy [28].

In endodontics, the used NaOCl concentration ranges between 0.5 and 6% [62]. Higher concentrations are more effective in necrotic tissue dissolution, leading to enhanced microbial eradication [29]. The current study used 5.25% NaOCl for the positive control group as it was proven to possess the most efficient antimicrobial ability and is therefore used in endodontic treatment [60,63,64]. While strong antimicrobial properties are crucial, it is essential to avoid damage to organic tissue if possible. All tested concentrations of NaOCl were reported in the literature to reduce cellular viability [29]. Sodium hypochlorite accidents have also been reported [13,16,17], with effects ranging from mild discomfort to tissue necrosis [12,15,59,65] due to NaOCl’s nonspecific proteolytic action [66]. Although lower concentrations of NaOCl were shown to have a reduced cytotoxic effect on dental pulp stem cells [15], the disinfection potential and antimicrobial effect, particularly against resistant species, were not as satisfactory, which explains the tendency to use higher NaOCl concentrations [24,67]. The results demonstrate the biocompatibility of CM nano-irrigant; however, they also present some degree of morphological alteration. These changes are directly correlated with increasing concentrations and exposure time (Figure 1, Figure 2 and Figure 3) and should further be evaluated to understand the extent of possible tissue damage. Dental mesenchymal stem cells (DMSCs) are multipotent stem cells with similar potential to bone marrow-derived mesenchymal stem cells (BMMSCs) [67]. Despite their distinct in situ localization, these two cell types share several common characteristics. Factors known to regulate the proliferation and differentiation of odontoblast precursors have also been confirmed to influence osteoblastic differentiation and development. Bone formation regulators such as transforming growth factor-beta (TGF-β) and both bone morphogenetic proteins (BMP-2 and BMP-4) have also been found to promote the development of odontoblasts [68]. Further studies have proven that BMMSCs can differentiate into both epithelial and mesenchymal lineage cells, validating their use in dental research and tissue engineering [69]. Primary hBMMSCs are considered a better option for in vitro investigations, yet an immortalized cellular lineage has been utilized, as it is easier to obtain and maintain in unlimited amounts [70].

The search for a disinfectant with similar potential but less detrimental side effects is a never-ceasing effort in endodontics. In this study, we examined the biocompatibility of previously optimized concentrations of a CM nano-irrigant on the morphology, viability, proliferation, migration, and healing capability of hBMMSCs. The model used in this study was a telomerized human bone marrow-derived mesenchymal stem cell line, which was generated via the overexpression of the human telomerase reverse transcriptase gene. This cell line exhibits all the genetic markers and characteristic features expressed by primary hBMMSCs, including proliferation, differentiation, and multipotency [70,71]. When compared to dental pulp stem cells, they show similar gene expressions, proliferative ability, differentiation, and fibroblast-like morphology [72].

The use of nanocarriers can protect herbal medicines from degradation, oxidation, and enzymatic breakdown, preserving their bioactivity [73]. The biocompatibility of CS and liposomes has been previously discussed and established in the literature, and their clinical applications were mentioned in different fields [43,74,75,76,77]. Liposomes are widely used due to their practicality, cost-effectiveness, biocompatibility, and low cytotoxicity [78]. They have been used as drug carriers due to their high encapsulation efficiency and stability [74,79,80], enabling more targeted and efficient site delivery [81]. CS is a naturally occurring polysaccharide with confirmed in vivo biocompatibility to vital tissues [73,78,82]. CS-coated liposomes (chitosomes) have been designed to overcome the limitations of conventional liposomes through structural modifications [83,84,85]. They exhibited a positively charged surface that could interact with the negatively charged cell membrane, making them efficient nanocarriers for herbal medicine [86]. Therefore, chitosomes have emerged as a promising delivery carrier for myrrh herbal extract [87].

In this study, a group of unloaded chitosomes was investigated to confirm its inertness (Figure 1, Figure 2, Figure 3, Figure 4, Figure 5 and Figure 6). Although the proliferative rates were somewhat slower (Figure 4), the final result is relatively similar to the proliferative score of the negative control group (untreated cells with media). This aligns with previous reports in the literature that prove chitosan had no or minimal negative effect on cellular viability and proliferation [88,89]. Due to the original sources of both liposomes and chitosan, biocompatible results are not surprising [52,53,78,82].

The biocompatibility assays performed in this investigation aimed to evaluate the safety of the irrigant for stem cells in endodontic therapy applications. Microscopic images revealed minor morphological changes, particularly in the high-concentration group G3 (35 mg/mL). However, these changes do not appear to be harmful to the cells. This observation was further confirmed by immunofluorescence staining and imaging; DAPI/phalloidin staining showed intact, densely packed nuclei and abundant actin fibers (Figure 2). Viability and proliferation assays further supported these findings. AlamarBlue^TM^ assay confirmed the cellular viability in all experimental groups, although Group 3 (35 mg/mL) showed significantly fewer viable cells (Figure 3). None of the concentrations interfered with cellular proliferation over a 14-day observation period (Figure 4). However, Group 3, which had the highest concentration (35 mg/mL), demonstrated slightly slower progression. These findings indicate that cytotoxicity and interference with cellular viability and proliferation may occur at higher concentrations and with prolonged exposure periods.

A major limitation of the current study is that it was entirely conducted in vitro on isolated, cultured cells. This omits the effect of true biological interactions and defense mechanisms within a host and could render the attained results inconclusive. Another limitation is that the experimental solution was directly applied to the cells, whereas in clinical situations, the solution is delivered into the root canal, which is surrounded by dentin and could be extruded into the periapical area. Further studies are recommended in an ex-vivo model followed by in vivo testing to establish the biological interactive factors. Contact of the proposed material with the host tissue constitutes many variables, such as inflammatory cytokines and host tissue reaction.

Another limitation is the use of a hBMMSC model. Even though this model was previously used and justified for dental research [90,91], the use of stem cells of dental origin seems to be more relevant to the field.

Future investigations could evaluate the potential for specific types of differentiation, such as differentiation into odontoblastic and osteoblastic progenitor cells, following treatment with the *Commiphora molmol* nano-irrigant. The expression of specific odontogenic and osteogenic markers could also contribute to the further optimization and enhancement of the *Commiphora molmol* nano-irrigant.

## 4. Materials and Methods

The study protocol was reviewed and approved by the Institutional Review Board and the College of Dentistry Research Center at King Saud University (IRB No. E-21-5964 and CDRC No. PR 0134).

### 4.1. CM Nano-Irrigant Formulation

The oleo-gum resin of *Commiphora molmol* was purchased from a known local herbalist (Naturespirit©, Riyadh, Saudi Arabia). The purchased resin was authenticated by the Natural Products Department in the College of Pharmacy, Al Maarefah University, Riyadh, Saudi Arabia (voucher specimen no. CME-1 was preserved). An ethanolic extract was attained, and initial sensitivity tests were conducted to determine the minimum inhibitory concentration (MIC) and minimum bactericidal concentration (MBC) for the ethanolic extract before proceeding to formulate the nano-irrigation solution In summary, 100 mg/mL of myrrh ethanolic extract was prepared and then serially diluted ten-fold; the MIC and MBC were then determined to be 25 and 30 mg/mL, respectively.

Following the MIC and MBC procedures, preliminary evaluations were performed to determine the most efficient nanocarrier to utilize and achieve the highest possible encapsulation of the myrrh extract. Various nanocarriers were tested, including chitosan (CS), solid lipid nanoparticles (SLNs), poly-lactic-co-glycolic acid nanoparticles (PLGA-NPs), and liposomes (Lipoid GmbH, Ludwigshafen, Germany). After pilot testing utilizing these different nanocarriers, the final formulation that showed the best stability and compatibility was myrrh-liposomes coated with chitosan (myrrh-chitosomes). CM-loaded chitosomes were prepared using lipoid S100 (10% *w*/*v*), CS (2 mg/mL), cholesterol (1% *w*/*v*), glycerol (1% *w*/*v*), and propylene glycol (1% *w*/*v*) in PBS (pH 7.4) according to previous studies [83,84,85]. The selected concentrations for loading the nanocarriers for biocompatibility evaluations were 25 mg/mL (MIC), 30 mg/mL (MBC), and 35 mg/mL. The experimental groups were G1 (MIC, 25 mg/mL), G2 (MBC, 30 mg/mL), G3 (35 mg/mL), positive control (5.25% NaOCl), and negative control (0.9% saline (NaCl)), and an additional group with the selected nanocarrier (chitosomes) was established to confirm that the unloaded chitosomes were inert. The prepared chitosomes were characterized with the values of 477.6 ± 19.8 nm, 34.9 ± 4.2 mv, and 0.451 ± 0.022 for particle size, zeta potential, and polydispersity index, respectively.

### 4.2. Cell Culture

A telomerized human bone marrow-derived mesenchymal stem cell line produced through the overexpression of the human telomerase reverse transcriptase gene (hTERT) was used in this study following previously described protocols [70,71]. The hMSC-TERT line was kindly provided by the Molecular and Cell Biology (MCB) Laboratory at the College of Dentistry in collaboration with the Prince Naif Bin Abdulaziz Health Research Center (King Saud University, Riyadh, Saudi Arabia). Human bone marrow-derived mesenchymal stem cell line (hMSC-TERT) cells were cultured in basal Dulbecco’s modified Eagle’s medium (DMEM, containing 4500 mg/L D-glucose, 4 mM L-glutamine, and 110 mg/L sodium pyruvate) supplemented with 10% fetal bovine serum (FBS), 1% MEM non-essential amino acids, and 1% antibiotic–antimycotic solution (all purchased from Gibco™, Thermo Fisher Scientific, Waltham, MA, USA). The flasks were maintained under aseptic conditions in a humidified incubator with 5% CO_2_ at 37 °C. The medium was changed twice weekly until a confluent cell monolayer formed. After which, the cells were detached with a 0.25% trypsin–EDTA solution (Gibco™), subcultured, counted, and resuspended for further experiments [71,92].

### 4.3. Cell Morphology

The morphology of the hMSC-TERT cells was monitored using an inverted Axiovert 40C Imaging Microscope (Carl Zeiss Microscopy GmbH, Göttingen, Germany), equipped with an EOS 700D digital camera (Nikon, Dusseldorf, Germany). Upon reaching 80% confluency, cells were exposed to different concentrations of CM nano-irrigant (unloaded, 25 mg/mL, 30 mg/mL, and 35 mg/mL) for 1 h, alongside positive and negative control groups, and the changes in spindle-shaped morphology were imaged and examined after 24–48 h [71,93].

### 4.4. Immunofluorescence Staining

The hMSC-TERT cells were seeded in 4-well dishes at 5 × 10^4^ cells/well and incubated with the various test concentrations of the CM nano-irrigant alongside the control groups for 1 h as previously described [93]. After 24 h of culture, the cells were washed twice with PBS (phosphate-buffered saline, Gibco™) and fixed with 4% paraformaldehyde in PBS (Sigma-Aldrich, St. Louis, MO, USA) for 20 min at 4 °C; the cell membrane was permeabilized for 15 min with 0.1% Triton X-100 (Sigma-Aldrich), and the cells were then treated with a blocking solution (10% bovine serum albumin (BSA) in PBS) for 1 h to block nonspecific binding sites. Thereafter, Alexa Fluor™ 594 Phalloidin (1:800, Invitrogen™ Molecular Probes, Eugene, OR, USA) was used to stain actin filaments for 20 min at room temperature, and 1 µM 4′,6-diamidino-2-phenylindole dihydrochloride (DAPI, Invitrogen™ Molecular Probes) was used to counterstain the nuclei for 10 min. All the samples were rinsed and stored in PBS before images were acquired with a Nikon C2 confocal laser scanning microscope (Nikon Instruments Inc., Tokyo, Japan) operated in the MCB Laboratory.

### 4.5. Cell Viability and Proliferation Assay

The cell viability and proliferation of hMSC-TERT cells were evaluated using an AlamarBlue^®^ assay (MyBioSource, San Diego, CA, USA), which quantifies cellular metabolic activity. A protocol previously described was followed with slight modifications [92]. Briefly, cells were seeded in 96-well culture plates at 1 × 10^4^ cells/well and incubated for 24 h to allow cell attachment. After cellular confluency was confirmed, the growth culture medium was aspirated from each well, and 100 µL of each CM nano-irrigant (unloaded, 25 mg/mL, 30 mg/mL, and 35 mg/mL) was added to each well and incubated for 1, 6, and 24 h. The wells containing 0.9% NaCl and 5.25% NaOCl were used as control groups, and 11 replicates were considered for each experimental condition. At the end of each incubation period, 20 µL of AlamarBlue^®^ reagent was added to the medium, and the plates were incubated for 4 h in the dark at 37 °C. The fluorescence signals were measured at an excitation wavelength of 530 nm and an emission wavelength of 590 nm with a Synergy II plate reader (BioTek Inc. Winooski, VT, USA). The cell viability relative to that of the control wells without nanoparticle exposure was calculated as [Cell viability, % = 100 × (FI 590 of the cells treated with nanoparticles/FI 590 of negative control cells)], where FI 590 = the fluorescent intensity at 590 nm emission (530 nm excitation).

For the proliferation assay, the cells treated with the *Commiphora molmol* nano-irrigant and 0.9% saline (negative control group) for 1 h were allowed to grow continuously in fresh culture media for 1, 3, 7, 10, and 14 days post-treatment. At the end of each exposure time, 10% AlamarBlue^®^ reagent was added to each well. The cells were incubated for 4 h in the dark, and the fluorescence (Ex 530 nm/Em 590 nm) was measured using a BioTek^®^ Synergy II microplate reader (BioTek Inc. Winooski, VT, USA).

### 4.6. Transwell Migration Assay

The 3D cell migration assay was performed using a transwell chamber (24 wells, 8 µm pore size; NEST Scientific, NJ, USA) as previously reported, with some modifications [92]. Briefly, hMSC-TERT cells were seeded at 5 × 10^5^ cells/well in the upper chambers with DMEM supplemented with 0.5% FBS, while the lower chambers were filled with 0.9% NaCl (negative control) or supplemented with different CM nano-irrigant concentrations for 1 h. To prevent cell proliferation, the migration assay was performed with fresh serum-free media for 24 h of incubation at 37 °C. Following the incubation period, the cells were fixed in 3.7% paraformaldehyde for 5 min and stained with 1% crystal violet (Sigma-Aldrich, Germany), followed by washing three times with PBS to remove the excess staining solution. The non-migrant cells were gently removed from the upper side of the membrane using a sterilized cotton swab. The number of migrated cells was quantified from ten random microscopic fields/inserts at 20× magnification with an inverted microscope. The cell number was counted, and the results from three independent experiments were averaged.

### 4.7. Wound-Healing Assay

Migration experiments were performed to quantify the wound-healing potential of treated hMSC-TERT cells when treated with the CM nano-irrigant at different concentrations. The initial cell density was 1 × 10^5^ cells/well in 12-well plates, and the cells were maintained at 37 °C until they had reached 90% confluence. A linear wound was made by scratching the monolayer of confluent cells at the bottom of the culture plate with a sterile 200 µL pipette tip. The debris was removed, and the cells were washed twice with PBS. The wound was treated with 0.9% NaCl solution (negative control) or with different CM nano-irrigant concentrations, and the plates were maintained at 37 °C with humidified 5% CO_2_ for 1 h. Finally, to quantify the cell migration rates at different time points (0, 24, and 48 h), the scratch-wound cell-free areas of each well were recorded using a phase-contrast microscope (Carl Zeiss Axiovert 40C Imaging Microscope) equipped with a digital camera. The rate of wound closure was calculated using the ImageJ software (version 1.50i, National Institutes of Health, MD, USA) as described previously [93].

### 4.8. Statistical Analysis

All the numerical data are reported as the means ± SDs (standard deviations), and images representing three independent experimental sets are provided. Statistical analysis was performed using either one-way or two-way analysis of variance (ANOVA) with GraphPad Prism 9 software (GraphPad Software, San Diego, CA, USA), followed by Tukey’s post hoc multiple comparison test, where the level of significance was set at *p* < 0.05.

## 5. Conclusions

In conclusion, the results affirm the biocompatibility of the CM nano-irrigant with hBMMSCs. Viability, proliferation, migration, and wound-healing assays showed positive results for all the experimental groups, with slightly decreased values in the higher-concentration Group 3 (35 mg/mL). These results highlight the potential of *Commiphora molmol* to be applied in endodontic treatment. However, further investigations should be conducted to overcome the limitations of in vitro studies and evaluate the in vivo effects and outcomes. The effects of *Commiphora molmol* on specific genetic markers involved in dentinogenesis and osteogenesis could further support or refute the current findings.

## Data Availability

The data presented in this study are available on request from the corresponding authors.
